# Supplementary material for: Application of the neuropeptide NPVF to enhance angiogenesis and osteogenesis in bone regeneration
Source: Commun Biol. 2023 Feb 20;6:197. doi: 10.1038/s42003-023-04567-x (PMC9941492; doi:10.1038/s42003-023-04567-x)
Supplement: Supplementary file 2 — Supplementary Information [file 42003_2023_4567_MOESM2_ESM.pdf]

Supplementary Information

Application of the neuropeptide NPVF to enhance angiogenesis and osteogenesis in bone regeneration

Hongping Yu[1 #], Yanyi Wang[2, 3, #], Junjie Gao[4,5], Youshui Gao[4, *], Chao Zhong[2, 3,*], Yixuan Chen[4,*]

1, Department of Orthopedic Surgery, The First Affiliated Hospital of Xiamen University, Xiamen, Fujian 361005, China

2, Center for Materials Synthetic Biology, Shenzhen Institute of Synthetic Biology, Shenzhen Institutes of Advanced Technology, Chinese Academy of Sciences, Shenzhen 518055, China

3, CAS Key Laboratory of Quantitative Engineering Biology, Shenzhen Institute of Synthetic Biology, Shenzhen Institutes of Advanced Technology, Chinese Academy of Sciences, Shenzhen 518055, China

4, Department of Orthopedic Surgery, Shanghai Jiao Tong University Affiliated Sixth People's Hospital, Shanghai 200233, China

5, Ningbo Institute of Life and Health Industry, University of Chinese Academy of Science, Ningbo, Zhejiang, China

# Hongping Yu and Yanyi Wang contributed equally to the manuscript.

Correspondence:

Youshui Gao, gaoyoushui@sjtu.edu.cn

Chao Zhong, chao.zhong@siat.ac.cn

Yixuan Chen, yixuanchen_sjtu@163.com

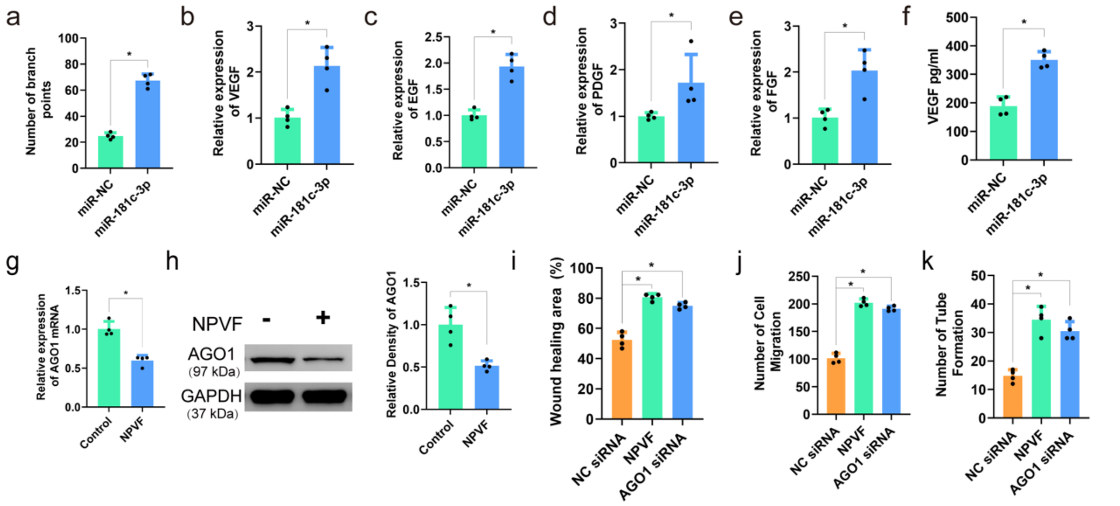

Fig. S1. Promotive effect of NPVF on HUVECs was mediated by miR-181c-3p. (A) Tube formation of HUVECs. Results were presented as means ± S.E.M. of four independent experiments, and the *P* value was 0.00006. $^{*}P < 0.05$, Student's *t*-test. (B-E) The gene expression levels of VEGF (B), EGF (C), PDGF (D), and FGF (E) in HUVECs treated with microRNA mimic. Results were presented as means ± S.E.M. of four independent experiments, and the *P* values from left to right were 0.0565, 0.0003, 0.0021, and 0.0057. $^{*}P < 0.05$, Student's *t*-test. (F) The protein expression level of VEGF HUVECs treated with microRNA mimic. Results were presented as means ± S.E.M of four independent experiments, and the *P* value was 0.0002. $^{*}P < 0.05$, Student's *t*-test. (G) The gene expression level of AGO1 in HUVECs treated with NPVF. Results were presented as means ± S.E.M. of four independent experiments, and the *P* value was 0.0004. $^{*}P < 0.05$, Student's *t*-test. (H) The protein expression level of AGO1 in HUVECs treated with NPVF. Results were presented as means ± S.E.M. of four independent experiments, and the *P* value was 0.0038. $^{*}P < 0.05$, Student's *t*-test. (I) Wound healing of HUVECs. Results were presented as means ± S.E.M. of four independent experiments, and the *P* values 0.0005 (left) and 0.0106 (right). $^{*}P < 0.05$, one-way ANOVA. (J) Transwell migration of HUVECs. Results were presented as means ± S.E.M. of four independent experiments, and the *P* values ware 0.0001 (left) and 0.0363 (right). $^{*}P < 0.05$, one-way ANOVA. (K) Tube formation of HUVECs. Results were presented as means ± S.E.M. of four independent experiments, and the *P* values are 0.0002 (left) and 0.0002 (right). $^{*}P < 0.05$, one-way ANOVA.

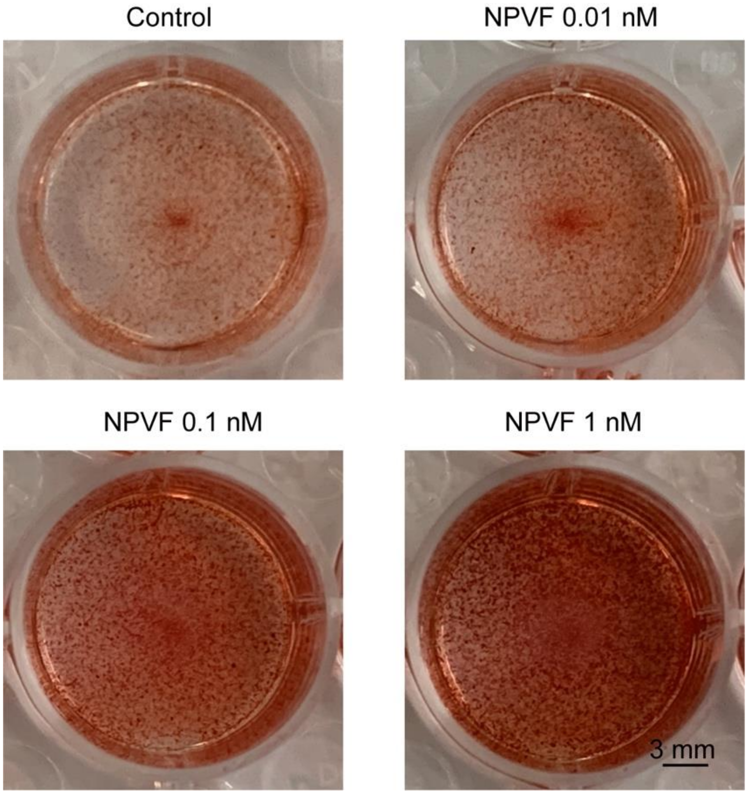

Fig. S2. NPVF-induced osteogenic differentiation of BMSCs visualized by Alizarin red staining. NPVF promotes the osteogenic differentiation of BMSCs in a dose-dependent manner.

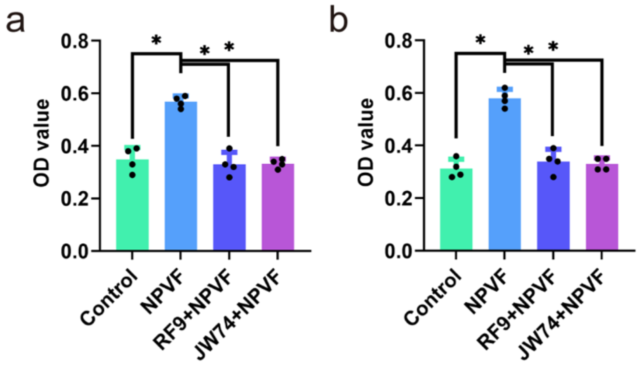

Fig. S3 The quantification of ARS and ALP staining. *p<0.05.

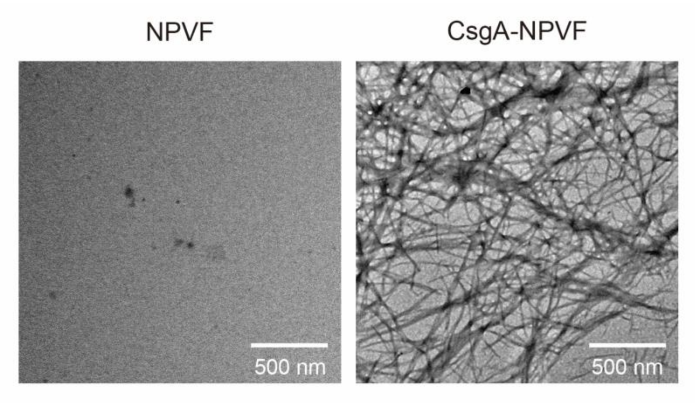

Fig. S4. TEM images of NPVF polypeptide and CsgA-NPVF nanofibers.

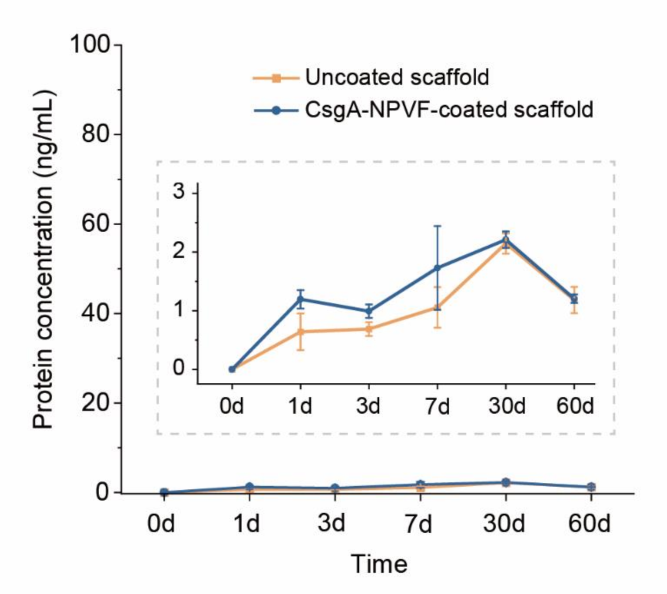

Fig. S5. Protein release curve of CsgA-NPVF-coated scaffolds and uncoated scaffolds in SBF solution.

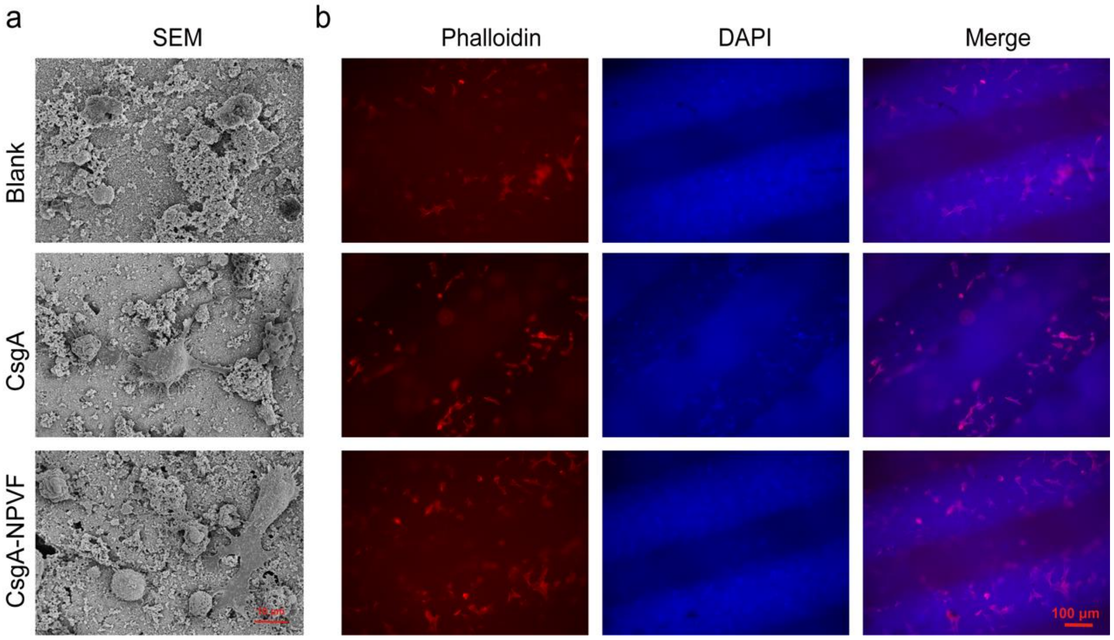

Fig. S6. Cytocompatibility of the CsgA-NPVF nanofiber coating. (A) SEM images of BMSCs on hydroxyapatite scaffolds with or without the CsgA-NPVF nanofiber coating. (B) Images of BMSCs adhering on calcium phosphate scaffolds with or without the CsgA-NPVF nanofiber coating stained with DAPI (nuclei, blue) and rhodamine phalloidin (cytoskeleton, red).

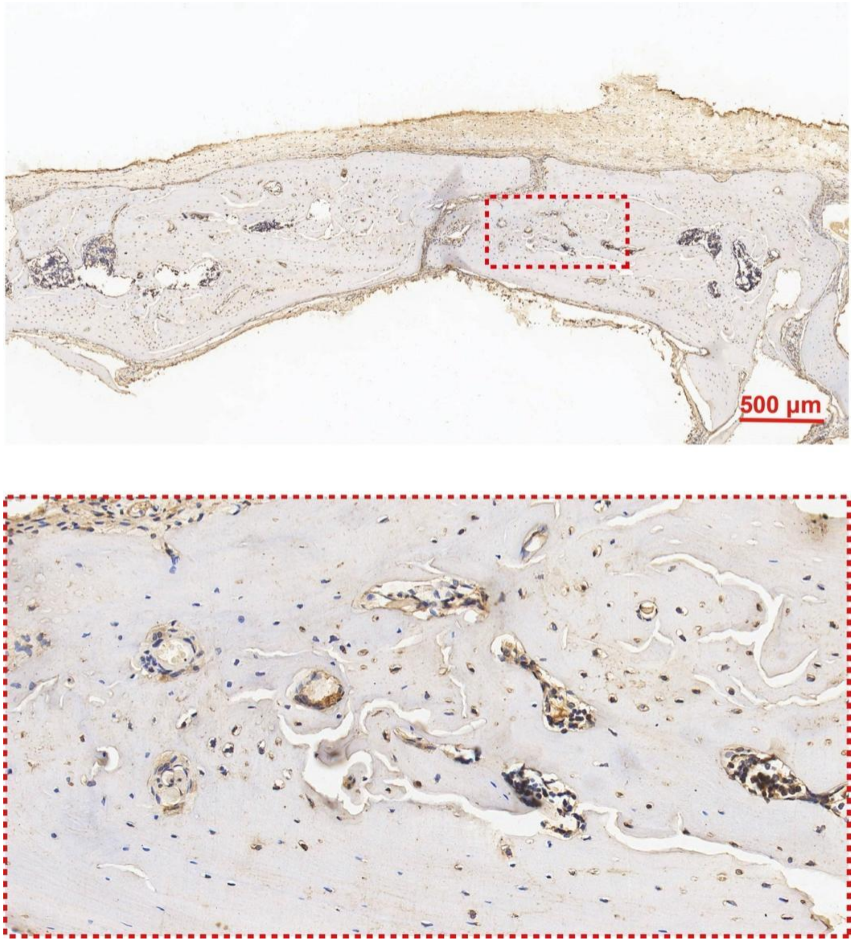

Fig. S7. Histological analysis of NPFFR1 staining in rat calvaria. NPFFR1 was highly expressed in rat calvarias.

Table S1. RT primers used in QPCR experiments.

Gene	Forward primer	Reverse primer
GAPDH	CCTTCCGTGTCCCCACT	GCCTGCTTCACCACCTTC
OPN	CTCCATTGACTCGAACGAC	GTGAAAACTTCGGTTGCTG
COL1	GACATCCCACCAATCACCTG	CGTCATCGCACAACACCTT
OCN	AGCCTTTGTGTCCAAGCA	CCAGCCATTGATACAGGTAG
Runx 2	GTAGATGGACCTCGGGAAC	TGCGCTACCTGAAACTGA
DLX5	CACCATCCGTCTCAGGA	CCATAGGAAGCCGAGGT
miR-181c-3p	GGAACCATCGACCGTTGA	-
U6	CTCGCTTCGGCAGCACA	-
NPFFR1	CCGGCTACAAACCTCACCTT	CAGTGTGAAAACGGAAGCCG

Table S2. Plasmids used in this study.

Plasmids	Construct details	Source
pET22b-*CsgA*	IPTG inducible CsgA expression from the pET22b backbone plasmid.	This study
pET22b-*CsgA-NPVF*	IPTG inducible CsgA-NPVF expression from the pET22b backbone plasmid.	This study

Fig. 3i

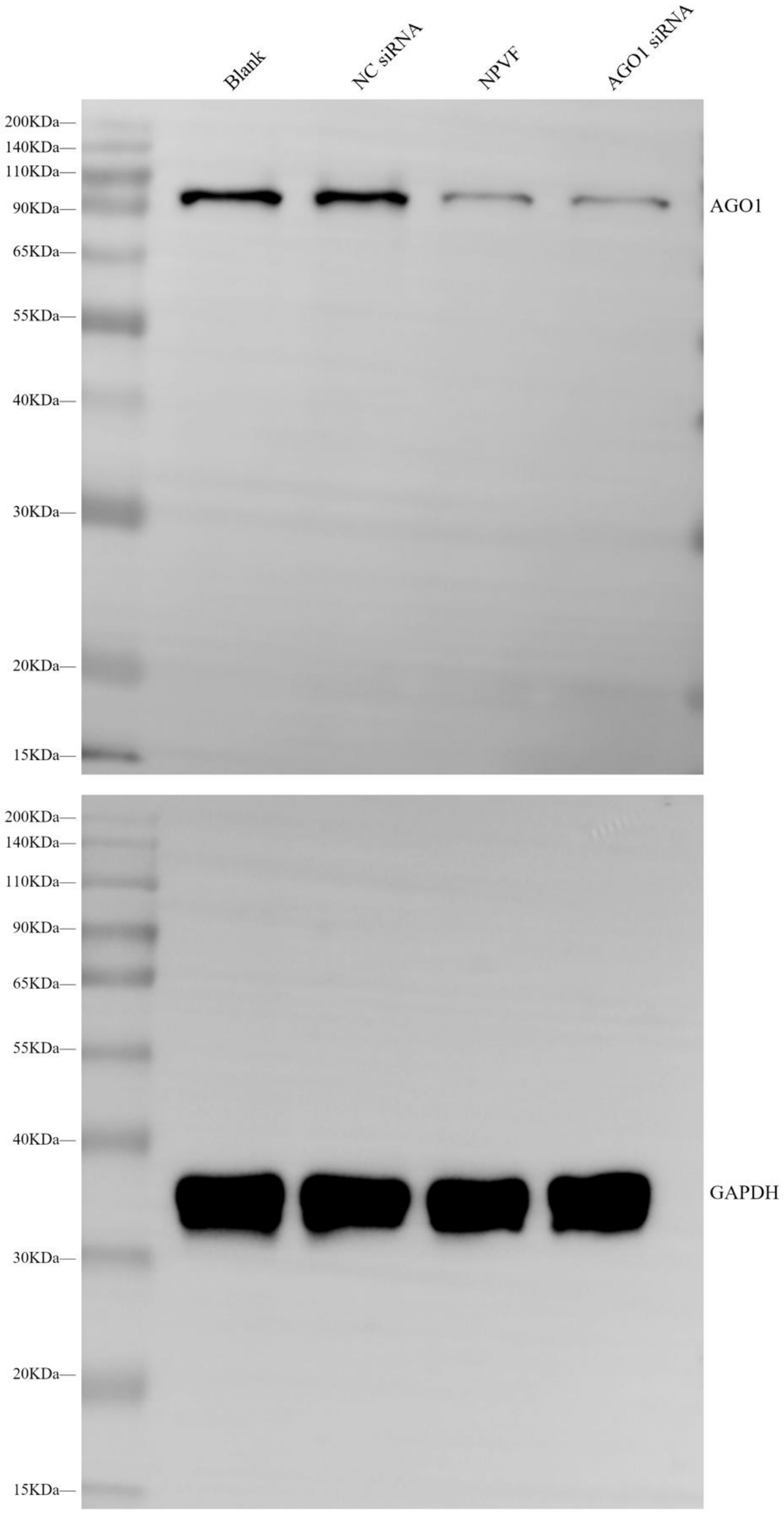
Blank
NC siRNA
NPVF
AGO1 siRNA
200KDa
140KDa
110KDa
90KDa
65KDa
55KDa
40KDa
30KDa
20KDa
15KDa
AGO1
200KDa
140KDa
110KDa
90KDa
65KDa
55KDa
40KDa
30KDa
20KDa
15KDa
GAPDH

Fig. 4c

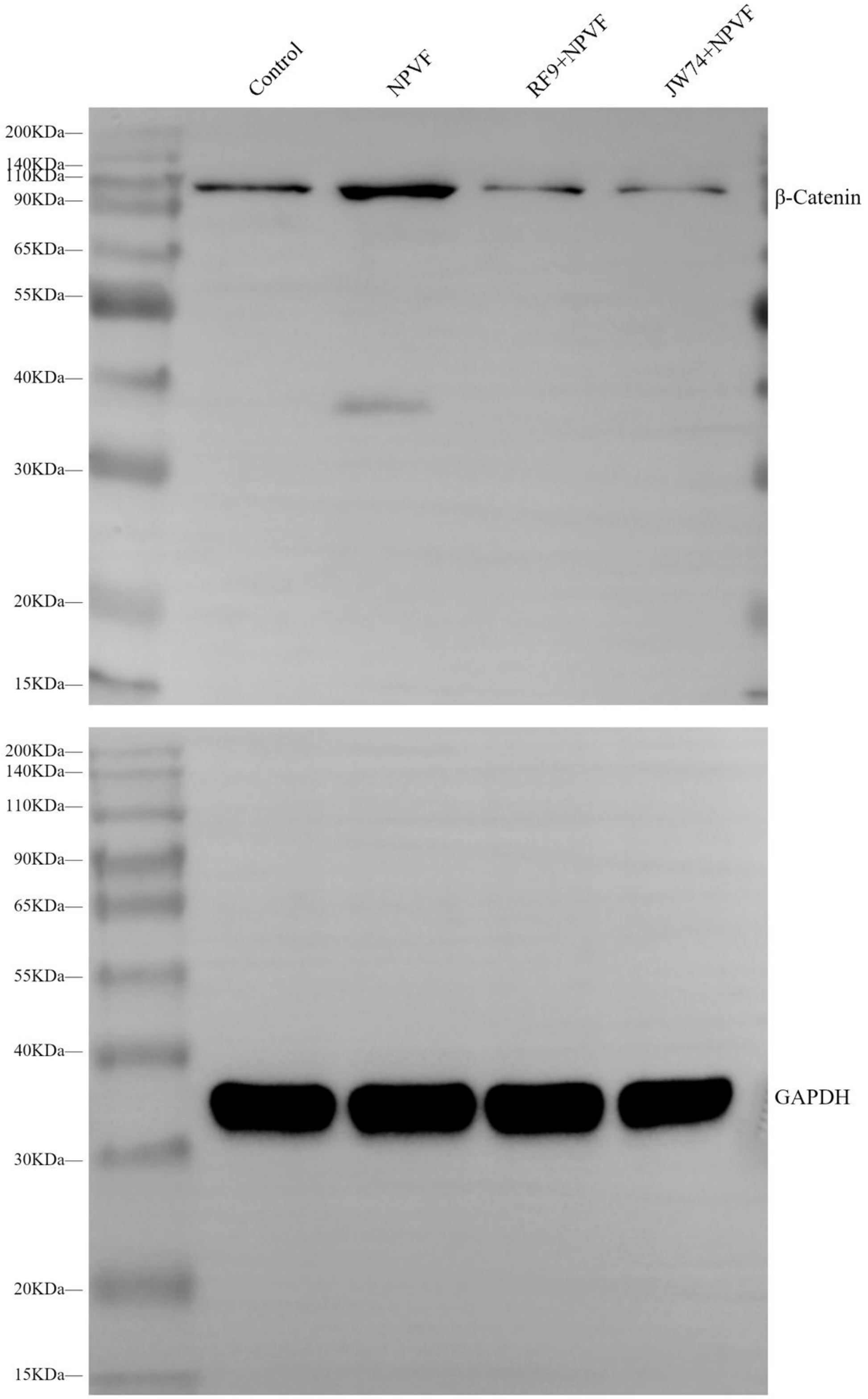
Control
NPVF
RF9+NPVF
JW74+NPVF
200KDa
140KDa
110KDa
90KDa
65KDa
55KDa
40KDa
30KDa
20KDa
15KDa
β-Catenin
200KDa
140KDa
110KDa
90KDa
65KDa
55KDa
40KDa
30KDa
20KDa
15KDa
GAPDH

Fig. S1h

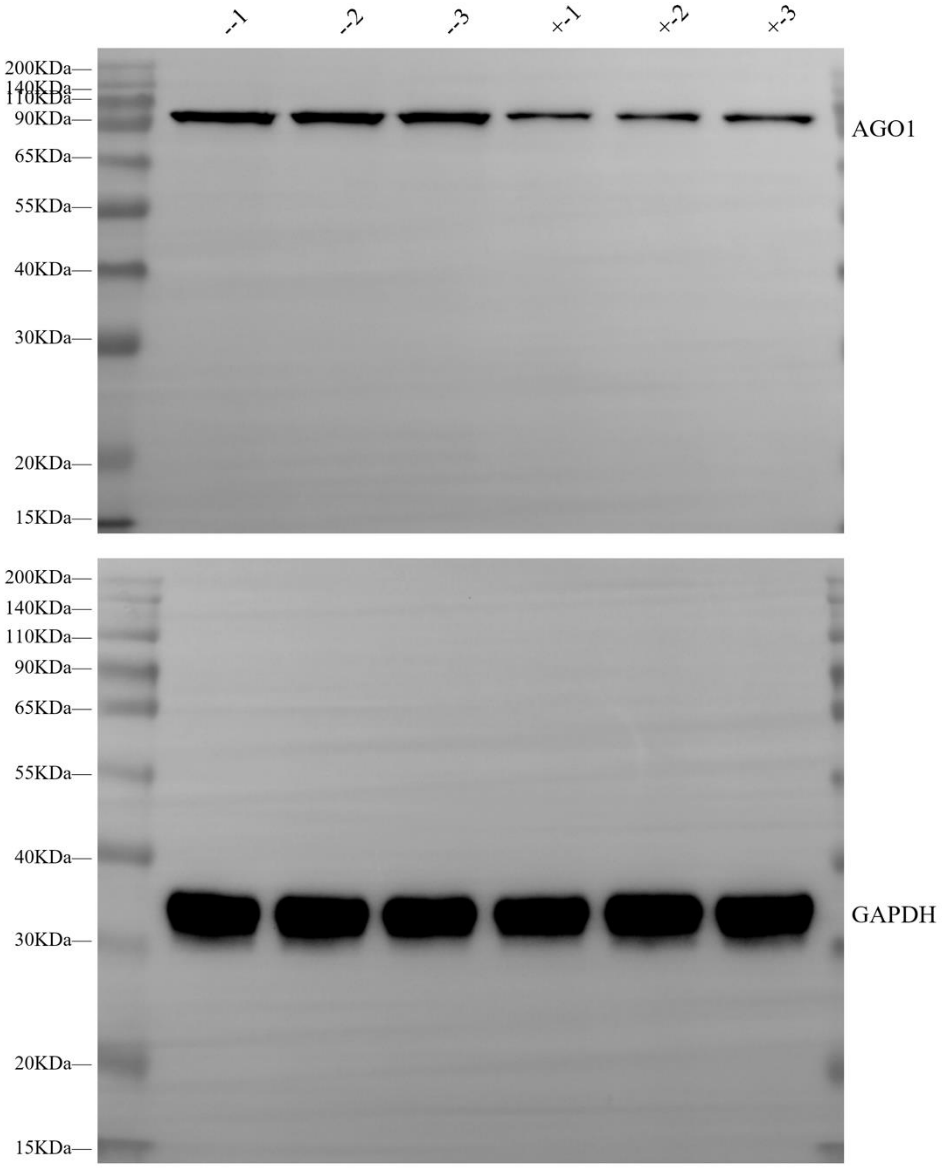

--1
--2
--3
+-1
+-2
+-3
200KDa—
140KDa—
110KDa—
90KDa—
65KDa—
55KDa—
40KDa—
30KDa—
20KDa—
15KDa—
AGO1
GAPDH
