# Supplementary material for: Application of the neuropeptide NPVF to enhance angiogenesis and osteogenesis in bone regeneration
Source: Commun Biol. 2023 Feb 20;6:197. doi: 10.1038/s42003-023-04567-x (PMC9941492; doi:10.1038/s42003-023-04567-x)
Supplement: Supplementary file 3 — Description of Additional Supplementary Files [file 42003_2023_4567_MOESM3_ESM.pdf]

Description of Additional Supplementary Files

File name: Supplementary Data
Description: The raw data supporting the figures of this study.
