# Supplementary material for: Application of the neuropeptide NPVF to enhance angiogenesis and osteogenesis in bone regeneration
Source: Commun Biol. 2023 Feb 20;6:197. doi: 10.1038/s42003-023-04567-x (PMC9941492; doi:10.1038/s42003-023-04567-x)
Supplement: Supplementary file 5 — Reporting Summary [file 42003_2023_4567_MOESM5_ESM.pdf]

nature portfolio

Corresponding author(s): Youshui Gao; Chao Zhong, Yixuan Chen

Last updated by author(s): Dec 30, 2022

Reporting Summary

Nature Portfolio wishes to improve the reproducibility of the work that we publish. This form provides structure for consistency and transparency in reporting. For further information on Nature Portfolio policies, see our Editorial Policies and the Editorial Policy Checklist.

Statistics

For all statistical analyses, confirm that the following items are present in the figure legend, table legend, main text, or Methods section.

n/a	Confirmed	
☐	☒	The exact sample size (n) for each experimental group/condition, given as a discrete number and unit of measurement
☐	☒	A statement on whether measurements were taken from distinct samples or whether the same sample was measured repeatedly
☐	☒	The statistical test(s) used AND whether they are one- or two-sided<br>*Only common tests should be described solely by name; describe more complex techniques in the Methods section.*
☒	☐	A description of all covariates tested
☒	☐	A description of any assumptions or corrections, such as tests of normality and adjustment for multiple comparisons
☐	☒	A full description of the statistical parameters including central tendency (e.g. means) or other basic estimates (e.g. regression coefficient) AND variation (e.g. standard deviation) or associated estimates of uncertainty (e.g. confidence intervals)
☐	☒	For null hypothesis testing, the test statistic (e.g. F, t, r) with confidence intervals, effect sizes, degrees of freedom and P value noted<br>*Give P values as exact values whenever suitable.*
☒	☐	For Bayesian analysis, information on the choice of priors and Markov chain Monte Carlo settings
☒	☐	For hierarchical and complex designs, identification of the appropriate level for tests and full reporting of outcomes
☒	☐	Estimates of effect sizes (e.g. Cohen's d, Pearson's r), indicating how they were calculated

Our web collection on statistics for biologists contains articles on many of the points above.

Software and code

Policy information about availability of computer code

Data collection	No special software code was used to collect data.
Data analysis	All data were plotted using the Graphpad prism 9.0.0 (121). We used Image J to analyze the western blot images. We used CTAn to analyze the BV/TV ratio and the BMD (g·cm-3) of the rat calvarias. We used Igor pro (version 6.12,W avemetrics Inc.) to analyze AFM raw data for better image presentation. Adobe illustrator (Version 2021) was used for pattern image processing.

For manuscripts utilizing custom algorithms or software that are central to the research but not yet described in published literature, software must be made available to editors and reviewers. We strongly encourage code deposition in a community repository (e.g. GitHub). See the Nature Portfolio guidelines for submitting code & software for further information.

Data

Policy information about availability of data

All manuscripts must include a data availability statement. This statement should provide the following information, where applicable:

- Accession codes, unique identifiers, or web links for publicly available datasets
- A description of any restrictions on data availability
- For clinical datasets or third party data, please ensure that the statement adheres to our policy

The main data supporting the findings of this study are available within the article and its supplementary information files. Extra data are available from the corresponding author upon reasonable request.

Field-specific reporting

Please select the one below that is the best fit for your research. If you are not sure, read the appropriate sections before making your selection.

☒ Life sciences ☐ Behavioural & social sciences ☐ Ecological, evolutionary & environmental sciences

For a reference copy of the document with all sections, see nature.com/documents/nr-reporting-summary-flat.pdf

Life sciences study design

All studies must disclose on these points even when the disclosure is negative.

Sample size	Samples sizes were decided based on preliminary experiments to provide sufficient power for statistical comparison. We used four replicate samples for cellular and molecular assays which could provide necessary statistical support; We used six replicate samples for Dual-luciferase reporter assay; We used five replicate samples for BMD and BV/TV analysis.
Data exclusions	No data were excluded from the analyses.
Replication	All experiments were independently repeated at least three times and all attempts to replicate the experiments were successful. We described details for each experiment in online method to reproduce all our experiments.
Randomization	Male Sprague-Dawley rats were acquired and randomly divided into three groups in this study.
Blinding	Investigators were not blinded during data collection and analysis because blinding would not provide any reliable datasets for our all biochemical and genetic experiments.

Reporting for specific materials, systems and methods

We require information from authors about some types of materials, experimental systems and methods used in many studies. Here, indicate whether each material, system or method listed is relevant to your study. If you are not sure if a list item applies to your research, read the appropriate section before selecting a response.

Materials & experimental systems

n/a	Involved in the study	
☐	☒	Antibodies
☒	☐	Eukaryotic cell lines
☒	☐	Palaeontology and archaeology
☐	☒	Animals and other organisms
☒	☐	Human research participants
☒	☐	Clinical data
☐	☒	Dual use research of concern

Methods

n/a	Involved in the study	
☒	☐	ChIP-seq
☒	☐	Flow cytometry
☒	☐	MRI-based neuroimaging

Antibodies

Antibodies used	Anti-GAPDH rabbit monoclonal antibody (CST, 5174, used at 1:1000 dilution)<br>AGO1 rabbit monoclonal antibody (CST, 5053, used at 1:1000 dilution)<br>β-catenin rabbit monoclonal antibody (CST, 8480, used at 1:1000 dilution)<br>HRP-conjugated Goat Anti-Rabbit igG (Servicebio, GB23204, used at 1:5000 dilution)
Validation	All antibodies have been extensively validated by the respective vendors: 1. https://www.cellsignal.cn/products/primary-antibodies/gapdh-d16h11-xp-rabbit-mab/5174?site-search-type=Products&N=4294956287&Ntt=gapdh&fromPage=plp. 2. https://www.cellsignal.cn/products/primary-antibodies/argonaute-1-d84g10-xp-rabbit-mab/5053?site-search-type=Products&N=4294956287&Ntt=ago1&fromPage=plp. 3. https://www.cellsignal.cn/products/primary-antibodies/b-catenin-d10a8-xp-rabbit-mab/8480?site-search-type=Products&N=4294956287&Ntt=catenin&fromPage=plp. 4. https://www.servicebio.cn/goodsdetail?id=263.

Animals and other organisms

Policy information about studies involving animals; ARRIVE guidelines recommended for reporting animal research

Laboratory animals	30 8-week-old male Sprague-Dawley rats

Wild animals	All rats were allowed to adapt one week before the experiment. Rats were killed under general anaesthesia.
Field-collected samples	All rats were sacrificed at week 12 and the calvarias were harvested. The rat skulls were scanned by a micro-CT scanner (Skyscan, Kontich, Belgium). Tomograms were reconstructed with the 3D creator software (Skyscan), and the CTAn image analysis software was used to calculate the BV/TV ratio and the BMD (g·cm-3) according to the reconstructed images. Next, the samples were decalcified, embedded, and cut (5-µm sections).
Ethics oversight	The animal experiment was approved by With the approval from the Animal Research Committee at Shanghai Sixth People's Hospital.

Note that full information on the approval of the study protocol must also be provided in the manuscript.

Dual use research of concern

Policy information about dual use research of concern

Hazards

Could the accidental, deliberate or reckless misuse of agents or technologies generated in the work, or the application of information presented in the manuscript, pose a threat to:

No	Yes	
☒	☐	Public health
☒	☐	National security
☒	☐	Crops and/or livestock
☒	☐	Ecosystems
☒	☐	Any other significant area

Experiments of concern

Does the work involve any of these experiments of concern:

No	Yes	
☒	☐	Demonstrate how to render a vaccine ineffective
☒	☐	Confer resistance to therapeutically useful antibiotics or antiviral agents
☒	☐	Enhance the virulence of a pathogen or render a nonpathogen virulent
☒	☐	Increase transmissibility of a pathogen
☒	☐	Alter the host range of a pathogen
☒	☐	Enable evasion of diagnostic/detection modalities
☒	☐	Enable the weaponization of a biological agent or toxin
☒	☐	Any other potentially harmful combination of experiments and agents
